# Supplementary material for: SPIRAL: integrating and aligning spatially resolved transcriptomics data across different experiments, conditions, and technologies
Source: Genome Biol. 2023 Oct 20;24:241. doi: 10.1186/s13059-023-03078-6 (PMC10590036; doi:10.1186/s13059-023-03078-6)
Supplement: Supplementary file 1 — Additional file 1: Figure S1. Simulating. A. Spatial arrangements of cell types on original and aligned coordinates by SPIRAL and PASTE for simulate 1-3 datasets. Colors represent cell types and shapes represent batches. B-C UMAP visualizations of simulate2-3 (B) and simulate4-5 (C) datasets, each dataset occupies two rows, in which colors represent cell types (upper) and batches (bottom). Figure S2. DLPFC. A. UMAP visualizations of four-section integrations for each donor (donor1 in upper, donor2 in middle, donor3 in bottom). B. Spatial arrangements of annotated layers on aligned coordinates by SPIRAL and PASTE (left, middle) and spatial distributions of clusters (mclust; right) on SPIRAL-aligned coordinates. C. Spatial distributions of raw, Seurat and SPIRAL-integrated domain-marker gene expressions, RORB, KRT17 and PCP4 in 151507, 151669, 151673 and SPIRAL-aligned coordinates. Figure S3. Sagittal mouse brain. A. H&E staining images of both anterior and posterior mouse brains. B. The anatomy diagram of sagittal mouse brain, where black line divides mouse brain into anterior and posterior parts. C. Spatial visualizations of annotated domain labels for four samples. D. UMAP visualizations of raw and integrated embeddings by five methods in integrating four samples. Each method occupies two columns, where domains are colored in left and batches are colored in right. E. Spatial arrangements of annotated domains on samples with one of replicates were rotated 60 degree. F. Spatial arrangements of annotated domains on aligned coordinates of anterior1&2 (left) and posterior1&2 (right) by SPIRAL (upper) and PASTE (bottom). G. Spatial disposition of mclust-derived clusters by SPIRAL on four samples. H. Comparison of the expressions of DEGs from cluster10 and DEGs from cluster 2,3,15,21 in mouse olfactory bulb domains. Figure S4. Coronal mouse brain. A. Annotations of domains generated by marker genes and Allen mouse brain atlas in three coordinates. B. UMAP visualizations o [file 13059_2023_3078_MOESM1_ESM.pdf]

Figure S1

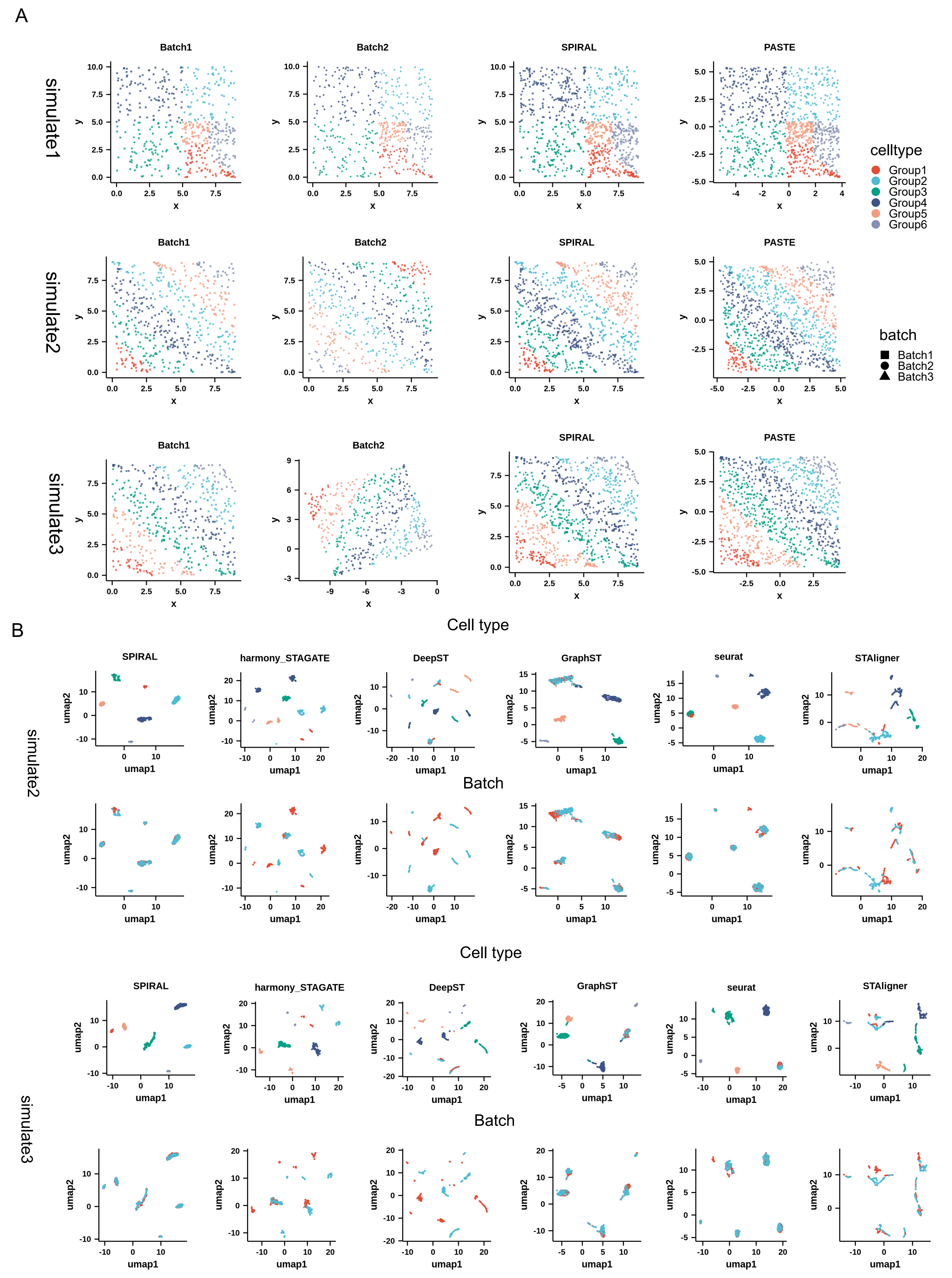
A
simulate1
simulate2
simulate3
Batch1
Batch2
SPIRAL
PASTE
x
y
celltype
Group1
Group2
Group3
Group4
Group5
Group6
batch
Batch1
Batch2
Batch3
B
Cell type
Batch
SPIRAL
harmony_STAGATE
DeepST
GraphST
seurat
STAligner
umap1
umap2

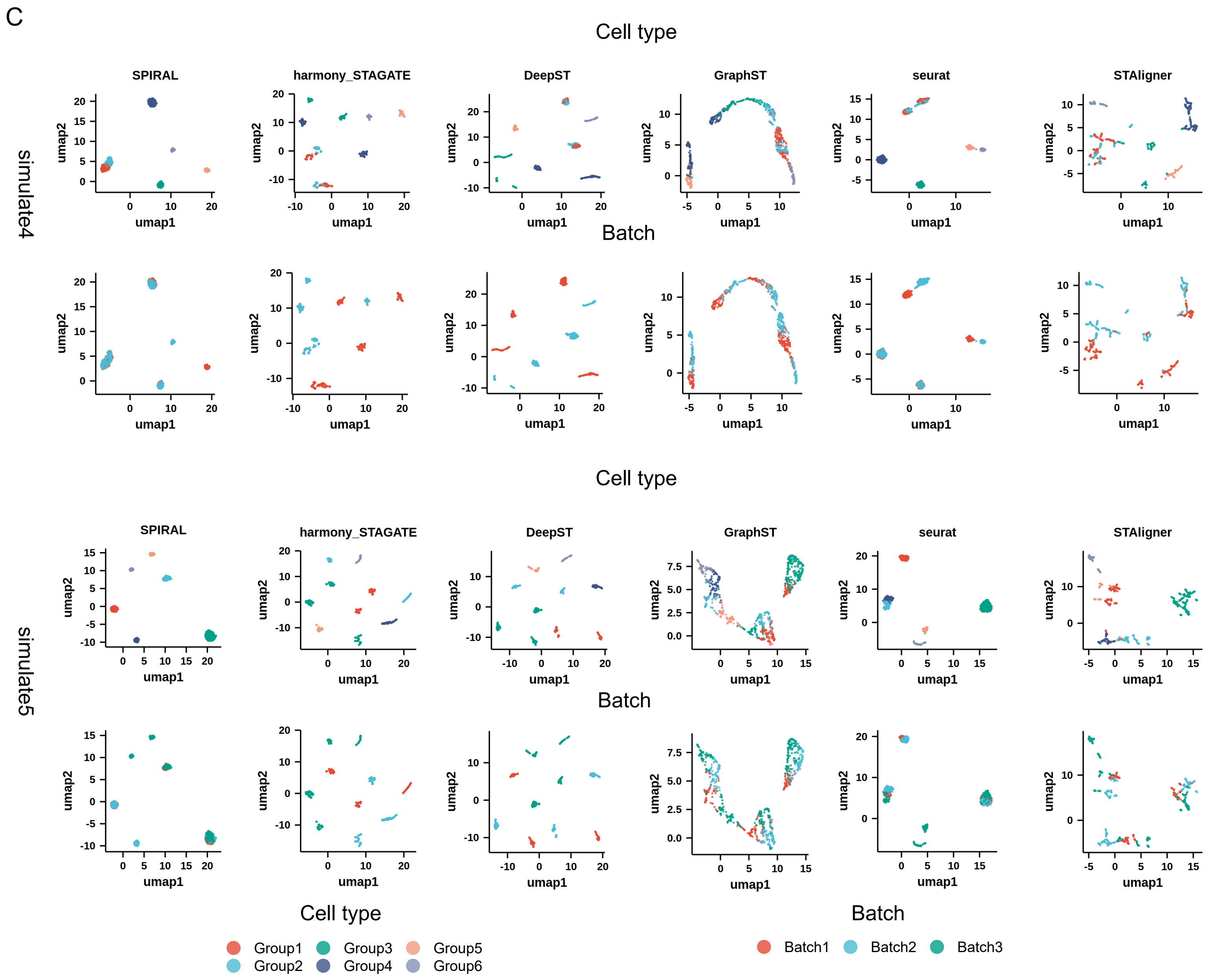

Figure S1: Simulating. **A** spatial arrangements of cell types on original and aligned coordinates by SPIRAL and PASTE for simulate 1-3 datasets. Colors represent cell types and shapes represent batches. **B-C** UMAP visualizations of simulate2-3 (B) and simulate4-5 (C) datasets, each dataset occupies two rows, in which colors represent cell types (upper) and batches (bottom).

Figure S2

A

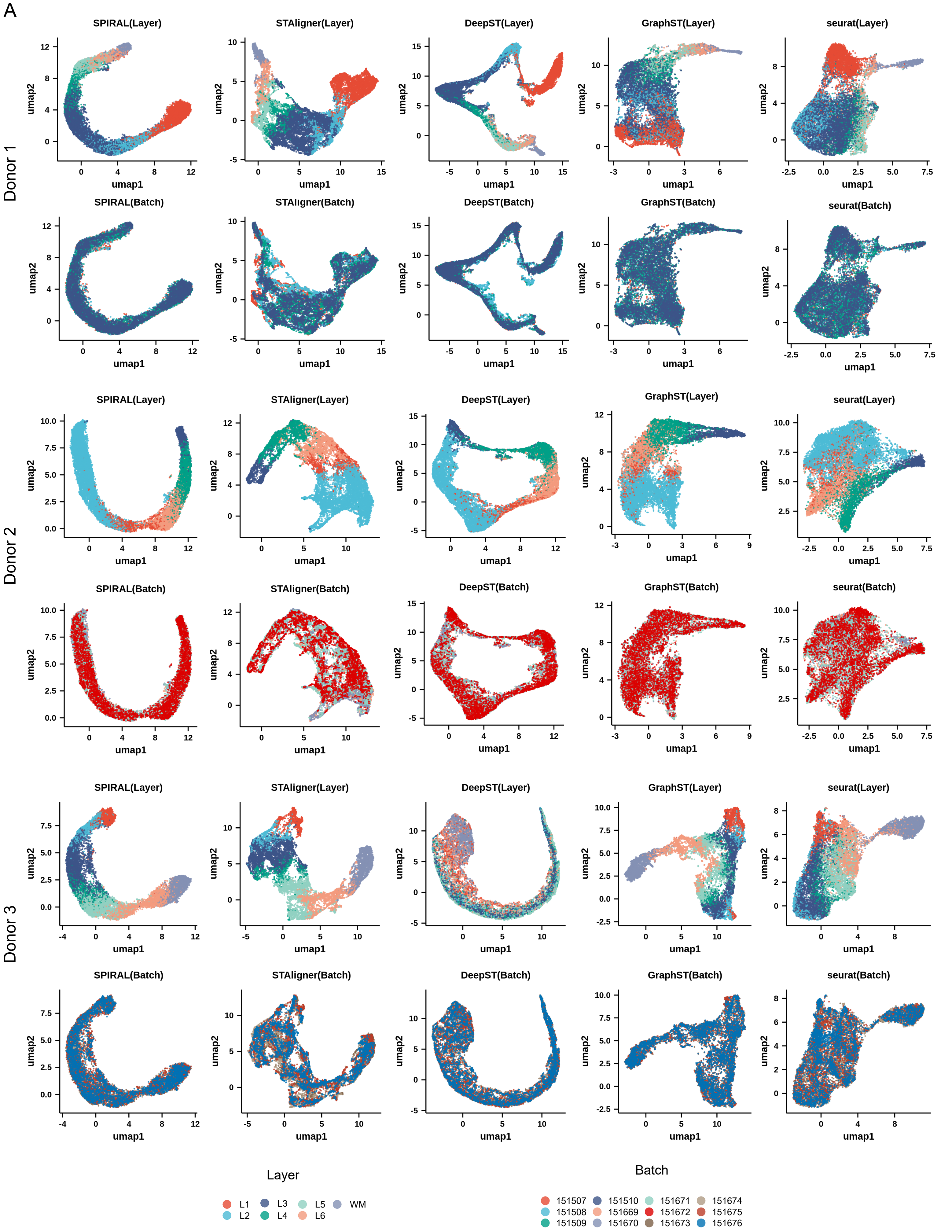
Donor 1
SPIRAL(Layer)
STAligner(Layer)
DeepST(Layer)
GraphST(Layer)
seurat(Layer)
SPIRAL(Batch)
STAligner(Batch)
DeepST(Batch)
GraphST(Batch)
seurat(Batch)
Donor 2
SPIRAL(Layer)
STAligner(Layer)
DeepST(Layer)
GraphST(Layer)
seurat(Layer)
SPIRAL(Batch)
STAligner(Batch)
DeepST(Batch)
GraphST(Batch)
seurat(Batch)
Donor 3
SPIRAL(Layer)
STAligner(Layer)
DeepST(Layer)
GraphST(Layer)
seurat(Layer)
SPIRAL(Batch)
STAligner(Batch)
DeepST(Batch)
GraphST(Batch)
seurat(Batch)
umap1
umap2
Layer
L1
L2
L3
L4
L5
L6
WM
Batch
151507
151508
151509
151510
151669
151670
151671
151672
151673
151674
151675
151676

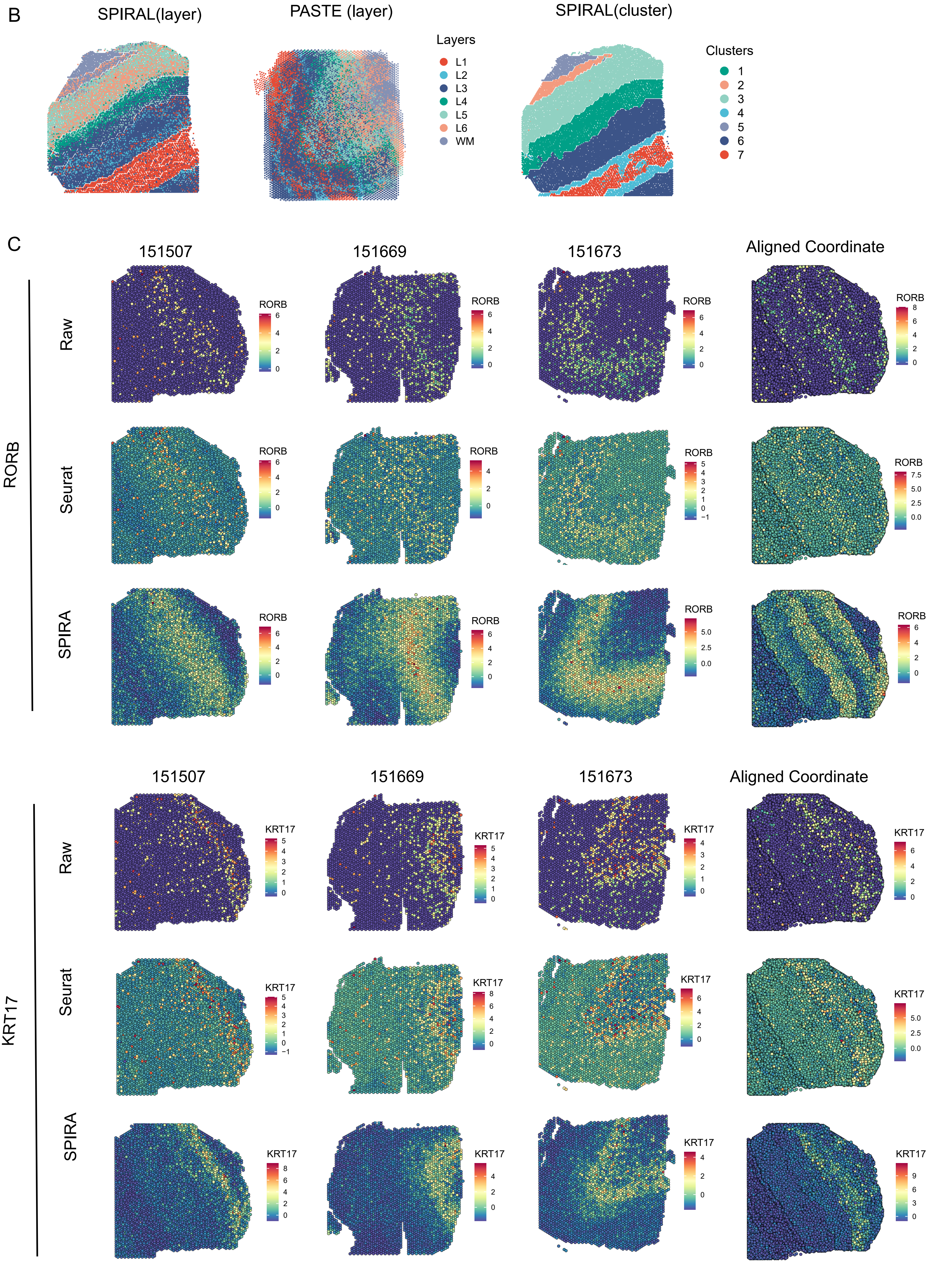
B
SPIRAL(layer)
PASTE (layer)
SPIRAL(cluster)
Layers
L1
L2
L3
L4
L5
L6
WM
Clusters
1
2
3
4
5
6
7
C
151507
151669
151673
Aligned Coordinate
RORB
Raw
Seurat
SPIRA
KRT17

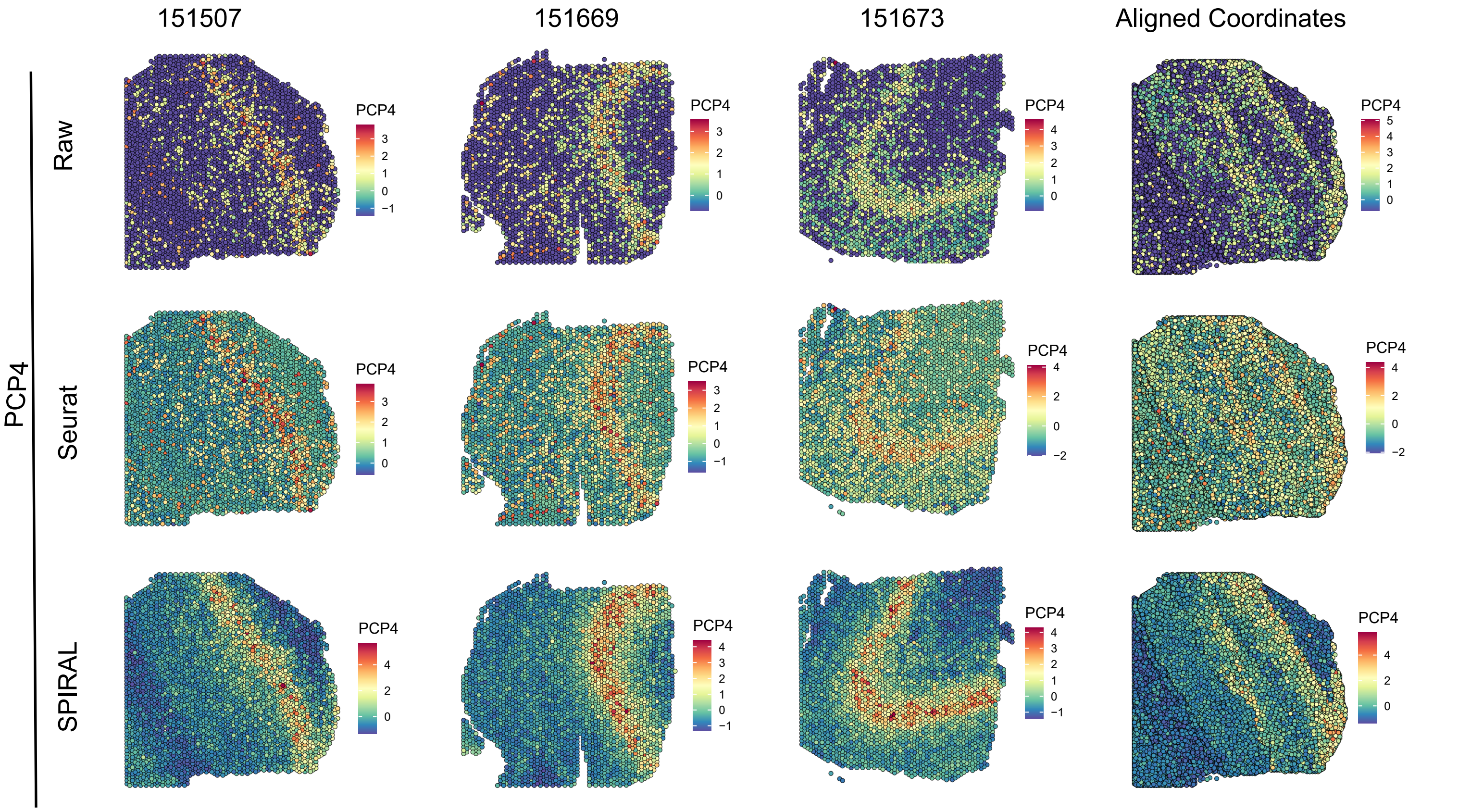

Figure S2: DLPFC. **A** UMAP visualizations of four-section integrations for each donor (donor1 in upper, donor2 in middle, donor3 in bottom). **B** Spatial arrangements of annotated layers on aligned coordinates by SPIRAL and PASTE (left, middle) and spatial distributions of clusters (mclust; right) on SPIRAL-aligned coordinates. **C** Spatial distributions of raw, Seurat and SPIRAL-integrated domain-marker gene expressions, RORB, KRT17 and PCP4 in 151507, 151669, 151673 and SPIRAL-aligned coordinates.

Figure S3

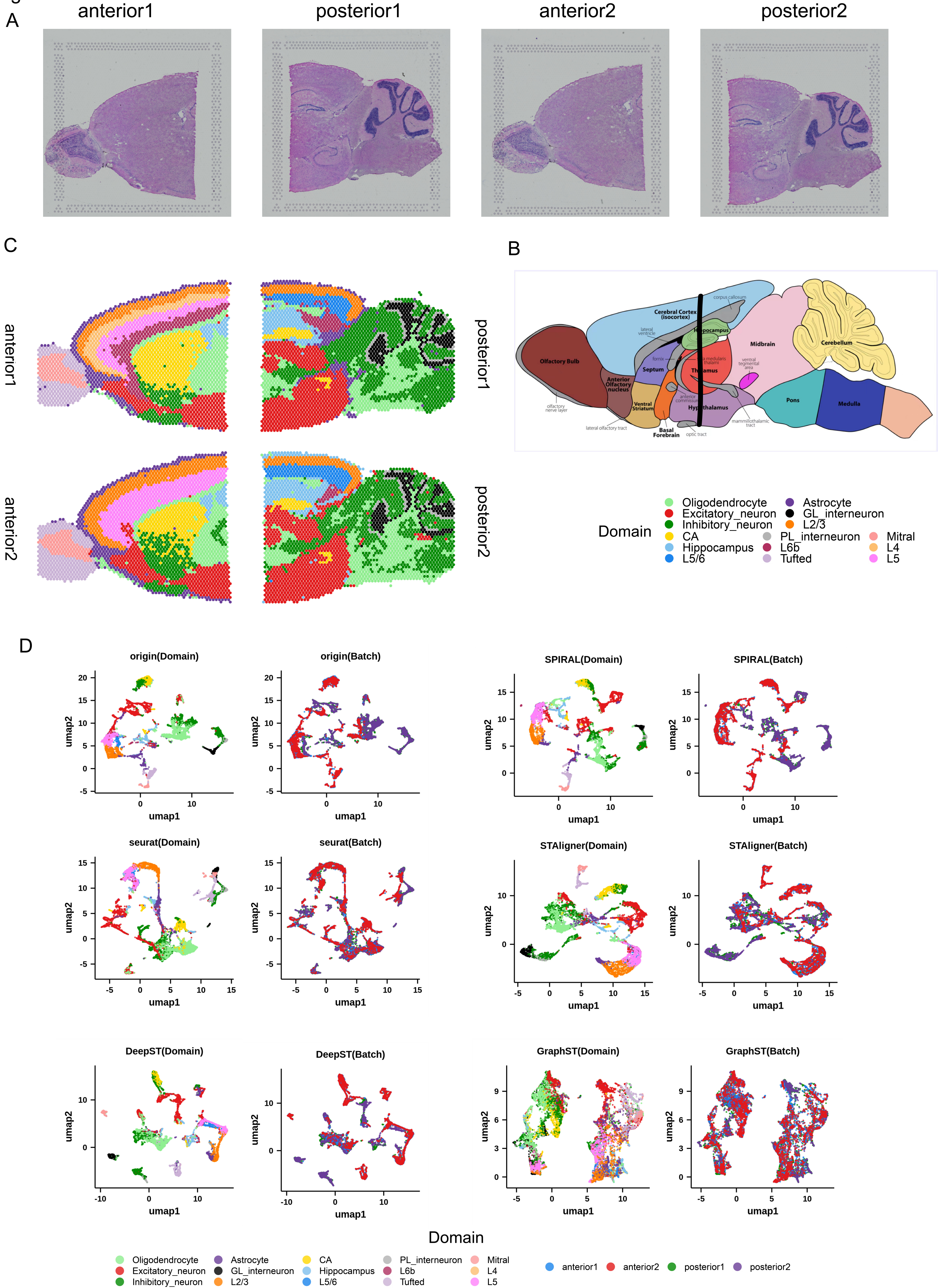
A
anterior1
posterior1
anterior2
posterior2
C
anterior1
posterior1
anterior2
posterior2
B
Cerebral Cortex (isocortex)
corpus callosum
Hippocampus
lateral ventricle
Midbrain
Cerebellum
Olfactory Bulb
Anterior Olfactory nucleus
Septum
fornix
Thalamus
ventral tegmental area
Pons
Medulla
Ventral Striatum
anterior commissure
Hypothalamus
mammillothalamic tract
olfactory nerve layer
lateral olfactory tract
Basal Forebrain
optic tract
Domain
Oligodendrocyte
Astrocyte
Excitatory_neuron
GL_interneuron
Inhibitory_neuron
L2/3
CA
PL_interneuron
Mitral
Hippocampus
L6b
L4
L5/6
Tufted
L5
D
origin(Domain)
origin(Batch)
SPIRAL(Domain)
SPIRAL(Batch)
seurat(Domain)
seurat(Batch)
STAligner(Domain)
STAligner(Batch)
DeepST(Domain)
DeepST(Batch)
GraphST(Domain)
GraphST(Batch)
umap1
umap2
Domain
Oligodendrocyte
Excitatory_neuron
Inhibitory_neuron
Astrocyte
GL_interneuron
L2/3
CA
Hippocampus
L5/6
PL_interneuron
L6b
Tufted
Mitral
L4
L5
anterior1
anterior2
posterior1
posterior2

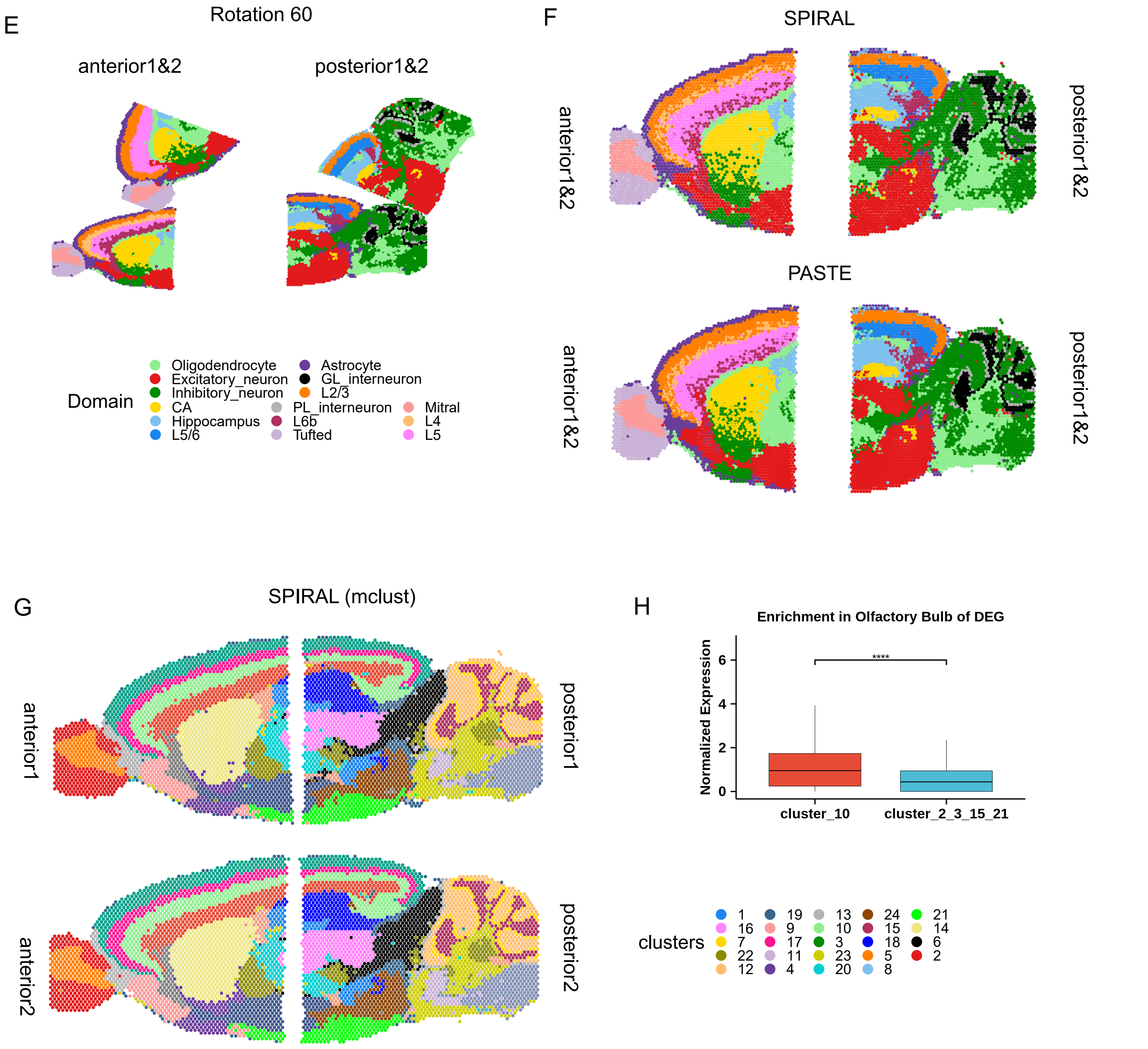

Figure S3: Sagittal mouse brain. **A** H&E staining images of both anterior and posterior mouse brains. **B** The anatomy diagram of sagittal mouse brain, where black line divides mouse brain into anterior and posterior parts. **C** spatial visualizations of annotated domain labels for four samples. **D** UMAP visualizations of raw and integrated embeddings by five methods in integrating four samples. Each method occupies two columns, where domains are colored in left and batches are colored in right. **E** Spatial arrangements of annotated domains on samples with one of replicates were rotated 60 degree. **F** Spatial arrangements of annotated domains on aligned coordinates of anterior1&2 (left) and posterior1&2 (right) by SPIRAL (upper) and PASTE (bottom). **G** Spatial disposition of mclust-derived clusters by SPIRAL on four samples. **H** Comparison of the expressions of DEGs from cluster10 and DEGs from cluster 2,3,15,21 in mouse olfactory bulb domains.

Figure S4

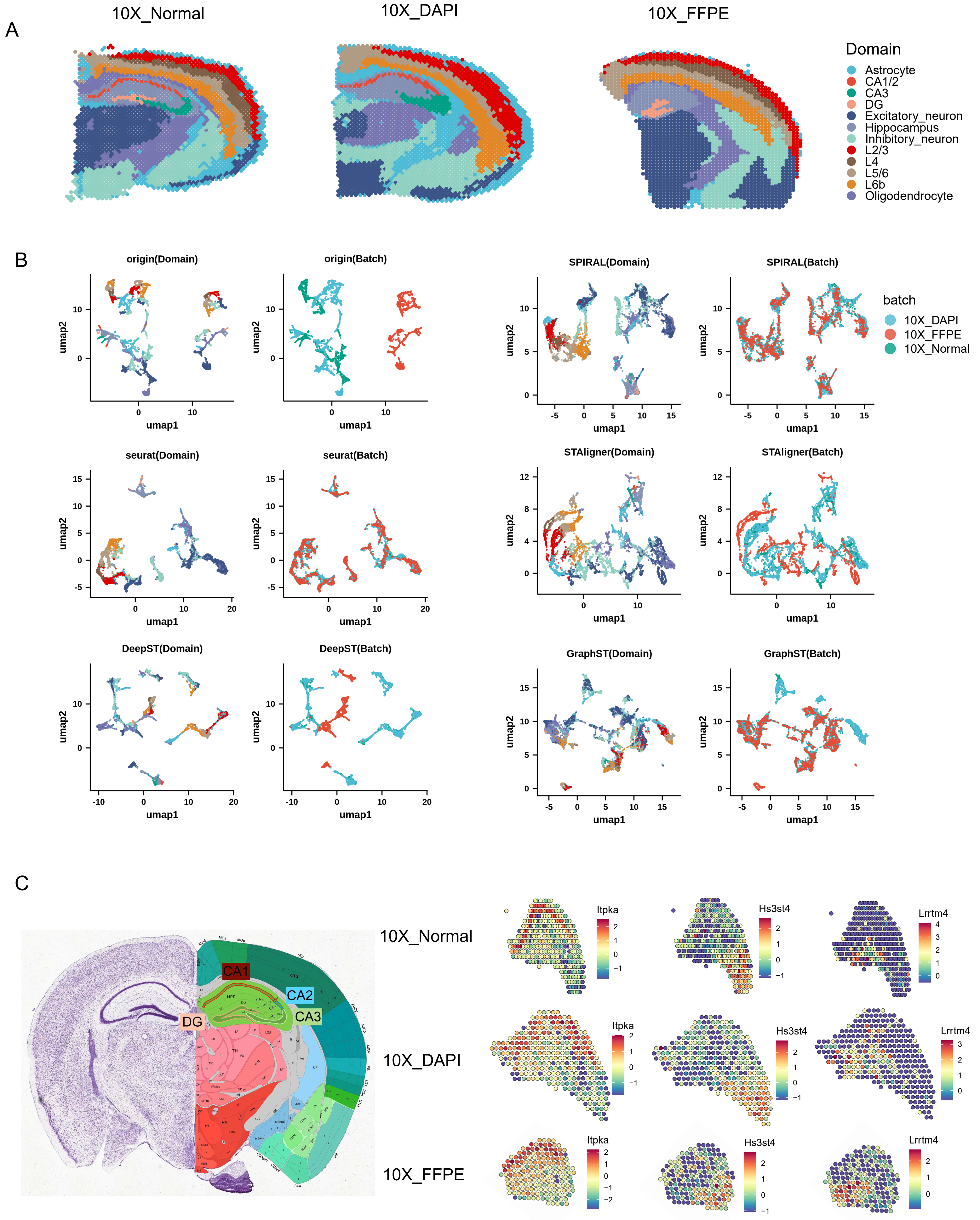
A
10X_Normal
10X_DAPI
10X_FFPE
Domain
Astrocyte
CA1/2
CA3
DG
Excitatory_neuron
Hippocampus
Inhibitory_neuron
L2/3
L4
L5/6
L6b
Oligodendrocyte
B
origin(Domain)
origin(Batch)
SPIRAL(Domain)
SPIRAL(Batch)
seurat(Domain)
seurat(Batch)
STAligner(Domain)
STAligner(Batch)
DeepST(Domain)
DeepST(Batch)
GraphST(Domain)
GraphST(Batch)
umap1
umap2
batch
10X_DAPI
10X_FFPE
10X_Normal
C
CA1
CA2
CA3
DG
10X_Normal
10X_DAPI
10X_FFPE
Itpka
Hs3st4
Lrrtm4

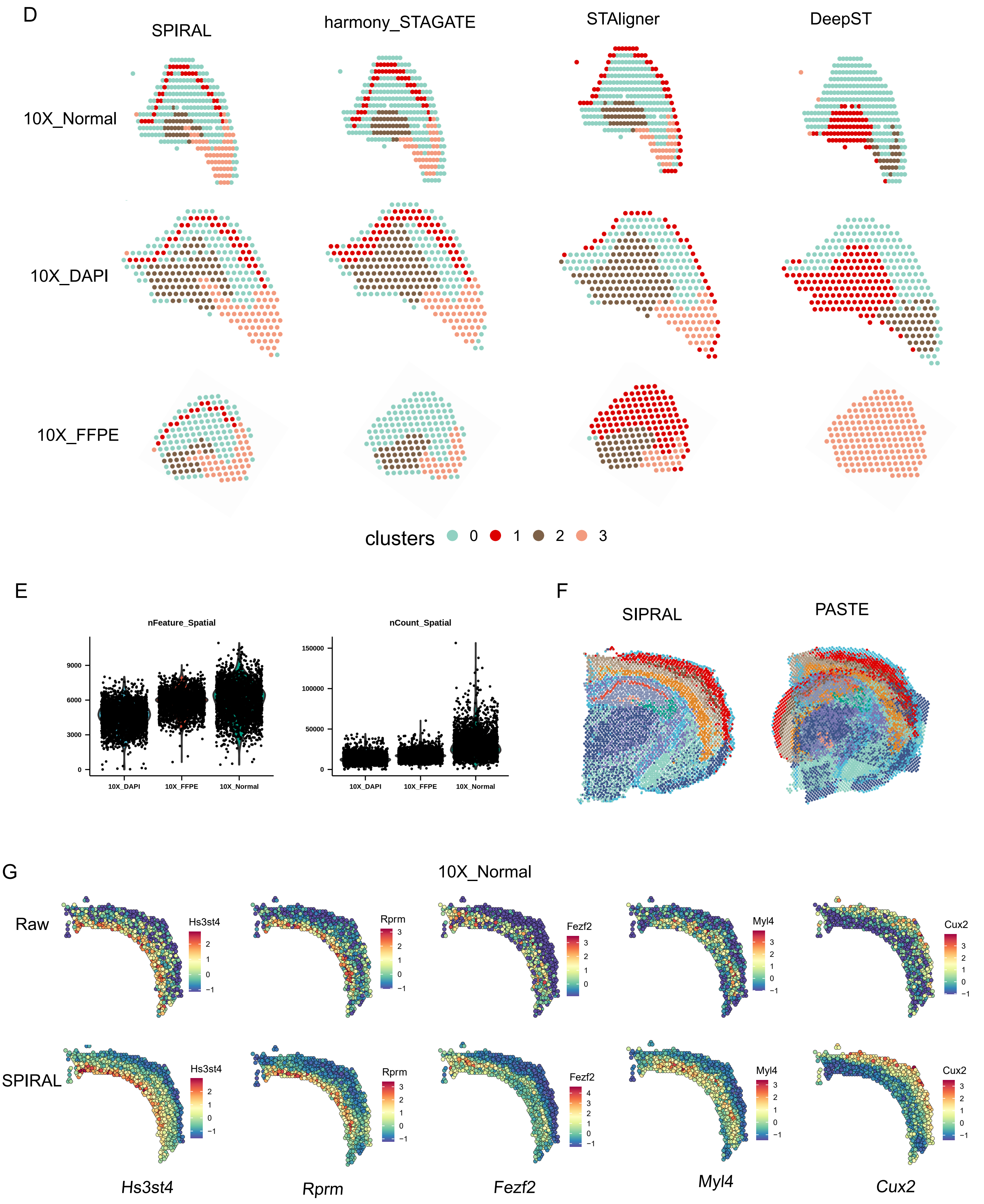

Figure S4: Coronal mouse brain. **A** Annotations of domains generated by marker genes and Allen mouse brain atlas in three coordinates. **B** UMAP visualizations of integrations of embeddings from origin and five methods. **C** The anatomy diagram of coronal mouse brain for hippocampus structures (left) and the distributions of the corresponding gene expressions (right). **D** The arrangements of derived clusters by for spatial based methods on the region of hippocampus. **E** Statistics of sequence depth of three protocols. **F** Spatial arrangements of annotated domains on aligned coordinates by SPIRAL (left) and PASTE (right). **G** Spatial patterns of domain-marker gene expressions from raw data and SPIRAL integrated data in the cortex region of 10X Normal data.

Figure S5

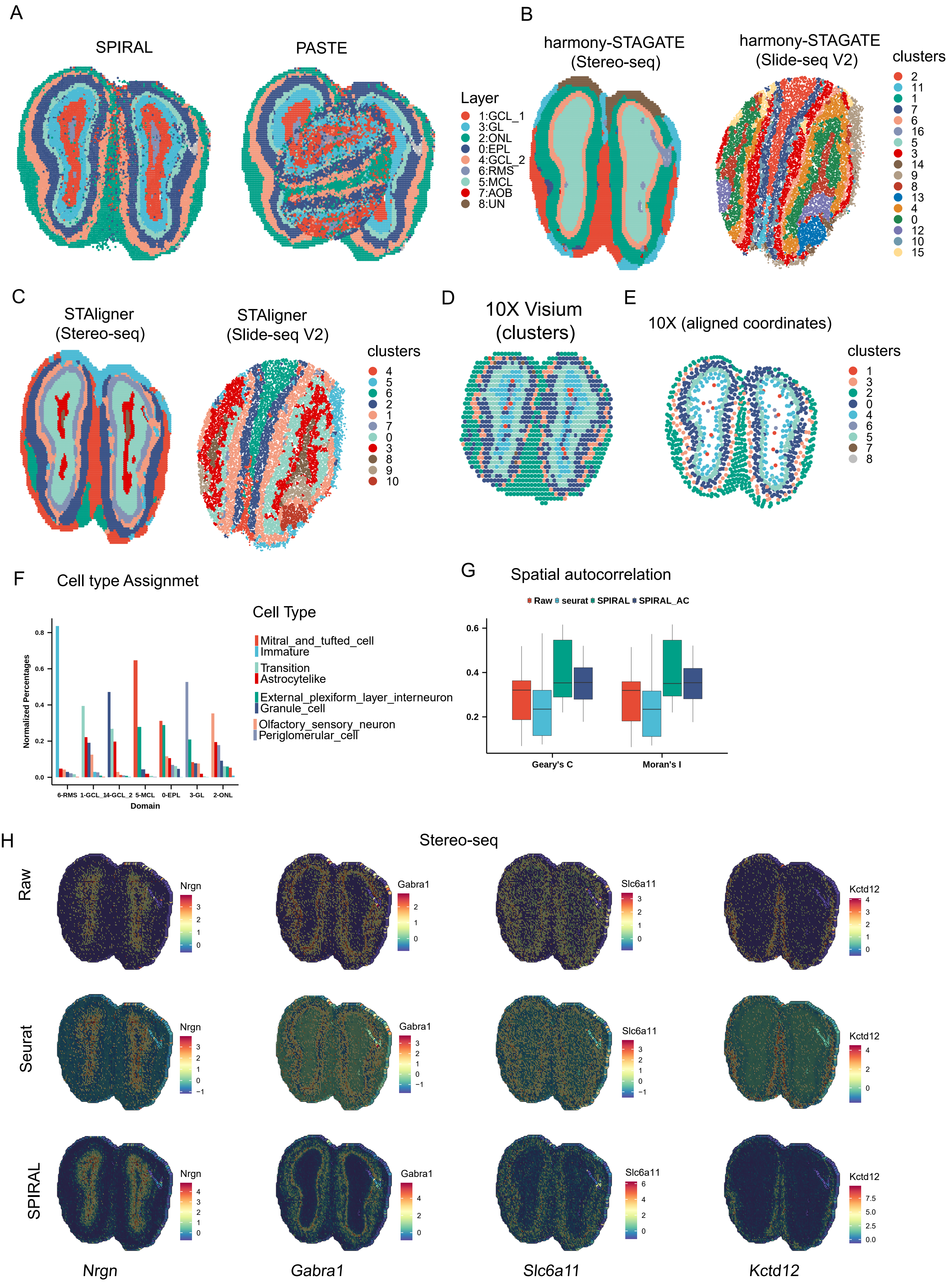
A
SPIRAL
PASTE
Layer
1:GCL_1
3:GL
2:ONL
0:EPL
4:GCL_2
6:RMS
5:MCL
7:AOB
8:UN
B
harmony-STAGATE (Stereo-seq)
harmony-STAGATE (Slide-seq V2)
clusters
C
STAligner (Stereo-seq)
STAligner (Slide-seq V2)
clusters
D
10X Visium (clusters)
E
10X (aligned coordinates)
clusters
F
Cell type Assignmet
Cell Type
Mitral_and_tufted_cell
Immature
Transition
Astrocytelike
External_plexiform_layer_interneuron
Granule_cell
Olfactory_sensory_neuron
Periglomerular_cell
Normalized Percentages
Domain
6-RMS
1-GCL_1
4-GCL_2
5-MCL
0-EPL
3-GL
2-ONL
G
Spatial autocorrelation
Raw
seurat
SPIRAL
SPIRAL_AC
Geary's C
Moran's I
H
Stereo-seq
Raw
Seurat
SPIRAL
Nrgn
Gabra1
Slc6a11
Kctd12

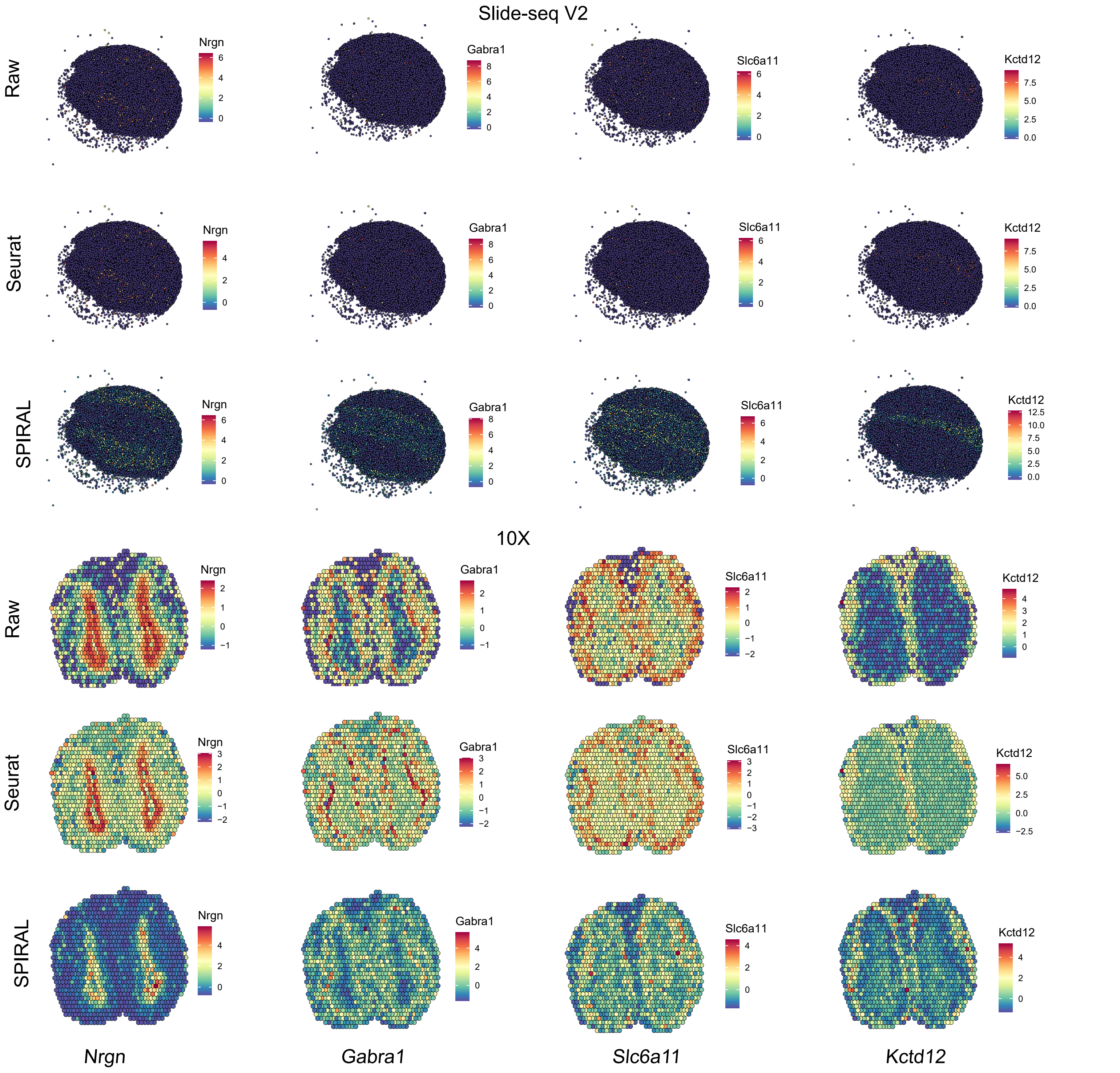

Figure S5: Mouse olfactory bulb. **A** Spatial distributions of clusters derived by SPIRAL in aligned coordinates by SPIRAL (left) and PASTE right). **B&C** Spatial distributions of Louvain-derived clusters on the embeddings of harmony_STAGATE (B) and STAligner (C). **D** Spatial distributions of predicted clusters of 10X Visium data using model trained by Stereo seq data and Slide V2 data. **E** Spatial distributions of of predicted clusters on aligned coordinates by SPIRAL. **F** The percentages of cell types in each cluster. **G** Spatial autocorrelations of raw and integrated gene expressions in original and aligned coordinates. **H** Spatial distributions of raw, SPIRAL-integrated and Seurat-integrated layer-marker expressions, Nrgn, Gabra1, Slc6a11 and Kctd12 on three samples. Each method occupies one row.
